# Thickness-dependent Crack Propagation in Uniaxially Strained Conducting Graphene Oxide Films on Flexible Substrates

**DOI:** 10.1038/s41598-017-02703-2

**Published:** 2017-06-01

**Authors:** Tushar Sakorikar, Maheswari Kavirajan Kavitha, Pramitha Vayalamkuzhi, Manu Jaiswal

**Affiliations:** 1 Department of Physics, Indian Institute of Technology Madras, Chennai, 600036 India; 2 Department of Electrical Engineering, Indian Institute of Technology Madras, Chennai, 600036 India

## Abstract

We demonstrate that crack propagation in uniaxially strained reduced graphene oxide (rGO) films is substantially dependent on the film thickness, for films in the sub-micron regime. rGO film on flexible polydimethylsiloxane (PDMS) substrate develop quasi-periodic cracks upon application of strain. The crack density and crack width follow contrasting trends as film thickness is increased and the results are described in terms of a sequential cracking model. Further, these cracks also have a tendency to relax when the strain is released. These features are also reflected in the strain-dependent electrical dc and ac conductivity studies. For an optimal thickness (3-coat), the films behave as strain-resistant, while for all other values it becomes strain-responsive, attributed to a favorable combination of crack density and width. This study of the film thickness dependent response and the crack propagation mechanism under strain is a significant step for rationalizing the application of layered graphene-like systems for flexible optoelectronic and strain sensing applications. When the thickness is tuned for enhanced extent of crack propagation, strain-sensors with gauge factor up to ∼470 are realized with the same material. When thickness is chosen to suppress the crack propagation, strain-resistive flexible TiO_2_- rGO UV photoconductor is realized.

## Introduction

Flexible electrodes and strain sensors have seen tremendous growth in their demand due to their ease of integration with non-conventional interfaces. In this regard, mainly two strategies have been adopted. One strategy relies on layered thin films cast on a flexible substrate, while an alternate strategy utilizes flexible composite films. While both have been studied extensively, each has its niche area of applications. For optoelectronic and device applications like solar cells^1^, transistors^2^, organic LEDs^3^, photodetector^4^, that necessitate energy band alignment, layered films have found favor due to the ease of interfacing. For applications in strain sensors, composites have shown more robust performance under mechanical deformation^5,6^ while layered films typically have relatively higher gauge factor. However, the success of these strategies highly depends upon their ability to withstand various forms of mechanically induced deformations like cracks and wrinkles. These deformations in layered morphology constitute as important attributes in flexible substrates and devices, and their effects have been studied extensively. Bowden *et al*.^7^ and others suggest methods to control the wrinkling/buckling of PDMS under oxygen plasma treatment, and thermal cycling on metal deposited substrates^8,9^. Conventional inorganic materials that have been employed as an electrode in such devices, have not been able to withstand strain more than 1.75%^10,11^, wherein the crack formation led to material failure, thus degrading the performance of the devices^12,13^. However, to develop commercially viable flexible devices, effect of cracks on electrode performance in such devices needs to be studied.

Graphene, an atomically thin 2-D membrane, due to its robust mechanical properties^14^ has been successfully shown to replace the existing conventional electrode materials in flexible devices^15–17^. Interestingly, when compared to pristine graphene, defected graphene was found to be more resistant to crack propagation, since the defects make the cracking process energetically less favorable^18^. Graphene oxide (GO) a chemically modified form of graphene, contains structural defects in the form of oxygen functional groups especially hydroxyls and epoxides which determine the crack formation and propagation^19^ and also the relative concentration of these groups controls mechanical failure of GO^20^. Thus one can tune the cracking of GO films by modifying the chemical composition which can be also achieved by reduction of GO to reduced graphene oxide (rGO), by chemical^21^ or thermal methods^22^. For commercial viability of rGO as an electrode material in flexible devices^3,23,24^ it is important to have an understanding of crack formation under strain and the limitations it imposes on electrical properties. Thomas *et al*. illustrated that buckling and cracking of GO films can be controlled by pre-straining the substrate^25^. Graphene layers have recently been used as a meta-interface to limit the cracking of Indium Tin Oxide (ITO) on polyethylene terephthalate (PET) substrates^26^. However, the interplay of strain-induced cracks, film thickness and the resultant strain-dependent electrical response of rGO on flexible substrates needs detailed investigation.

Here we report crack propagation and tunability in electrical response of conducting rGO films coated on PDMS under uniaxial strain. We show that by optimizing the thickness, rGO films can largely be made to retain their conductivity under strain up to 5%, a value which is well above the limit for conventional flexible electrode materials. Our study also reveals a facile way to achieve periodic cracking and control the crack density and crack width by varying the thickness. This is then utilized for strain sensing and also for strain resistant flexible photoconductor applications.

## Results and Discussions

**Figure 1:**
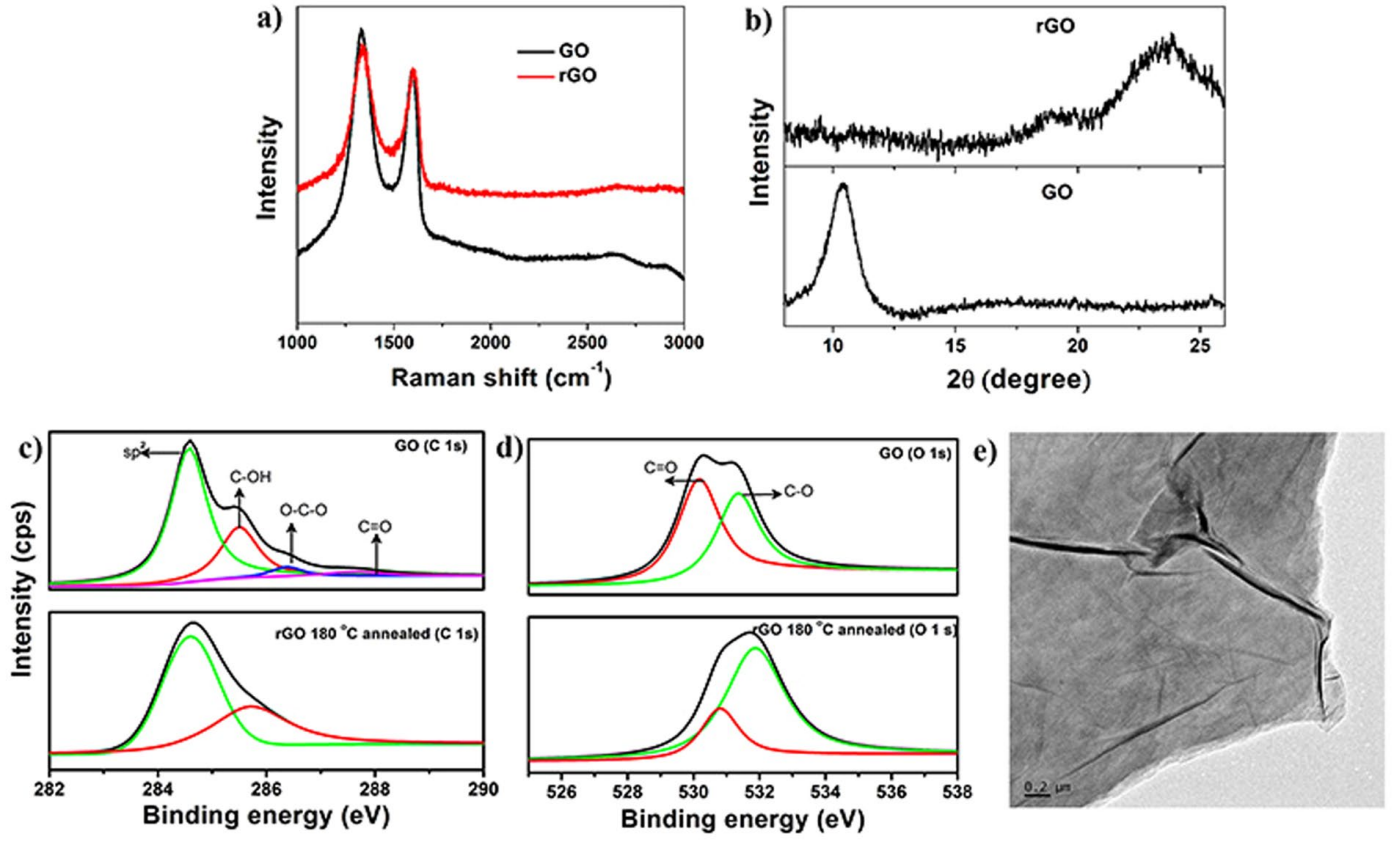
(**a**) Raman spectra of unstrained GO before and after annealing. (**b**) X-ray diffraction spectra of GO and rGO; High resolution (c) C1s and (d) O1s XPS spectra for GO and rGO. (**e**) TEM image of rGO film.

Figure 1a illustrates the Raman Spectra of the film, which shows the characteristic G-peak (at ~1578 cm^−1^), 2D-peak (at ~2700 cm^−1^) and D-peak (at ~1353 cm^−1^), for rGO. The D-peak is mainly due to the oxygen functionalities which are partially eliminated by annealing at 180 °C. Such defects can be reduced to a larger extent by annealing at high temperature (T ~ 800 °C)^27^, but here the polymer as a substrate presents as a limitation to the operational temperature that can be reached for annealing, subsequent to transfer of the film. However, the emergence of graphitic peak in the XRD spectra (Fig. 1b) shows that significant reduction is accomplished^28^. The C 1 s and O 1 s XPS spectra of GO and rGO annealed at 180 °C is shown in Fig. 1c and d respectively. The deconvoluted C 1 s spectra of GO consist of four components namely sp^2^ carbon (284.6 eV), C-OH (285.5 eV), O-C-O (286.6 eV) and a smaller component related to carbonyls at 288.0 eV. After annealing, C 1 s splits in to a doublet with major contribution from sp^2^ carbon peak indicating the restoration of sp^2^ domains. The O 1 s spectra of GO consist of doublet assigned to double bonded oxygen (530.5 eV) and single bonded oxygen from hydroxyls and epoxies (531.8 eV). After annealing O 1 s spectra becomes prominent with oxygen singly bonded to carbon due to the conversion of C = O to C-O during thermal reduction^29,30^. After annealing, increase in electrical conductivity by many orders in magnitude gives further evidence of the partial reduction of GO.

Figure 1e shows the transmission electron micrograph (TEM) of unstrained rGO films. The overlapping layered morphology with wrinkles and buckles can be clearly seen from the TEM image. Figure S1 shows the scanning electron microscope (SEM) image of unstrained rGO film on PDMS. Seemingly periodic box like patterns can be observed with wrinkles and buckles contained in them. These are ubiquitous in our samples and are contributed by two main reasons discussed as following. Oxygen-plasma treated PDMS films form buckling patterns with locally ordered and globally disordered waves^9^. This has been attributed to the reason when PDMS films are treated with oxygen plasma, an oxide layer formation takes place on its surface. This oxide layer has a lower thermal expansion coefficient (TEC) than the bulk PDMS. The spontaneous formation of wrinkled patterns on thermal cycling has been discussed in previous literature^7,9,31^. Furthermore, GO has a negative thermal expansion coefficient (TEC)^32^ while PDMS has positive TEC^7^. When this multilayer film with different TEC is subject to thermal cycling, compressive thermal stress due to TEC mismatch builds up between the layers. The heating cycle also involves removal of functional groups and expulsion of confined water layers^28^. The compressive stress is released in the cooling cycle in the form of permanent deformations (wrinkles) with wavelengths ranging from 3 to 12 μm^7,31^. These values for wrinkle wavelengths compare well with numerical estimates based on the elastic mismatch between the rGO and oxide layer (4.5 μm), as well as between the oxide layer and PDMS (7.5 μm). Secondly, a large number of sub-micron wrinkles that branch out from individual long wrinkles can be seen in the SEM image (Figure S1b) taken on a magnified scale. These have been attributed to the topological defects created by the removal of epoxy groups during thermal reduction of GO, which is accompanied by the release of CO_2_ gas^33^. When a uniaxial strain is applied on rGO films, these wrinkles play a critical role in resisting the deformations of films (Figure S2). It is further possible to tune the degree of buckling and wrinkling in the rGO film by suitably pre-straining the substrate before the rGO deposition^25^. In our work, rGO films are coated on unstrained substrates.

**Figure 2:**
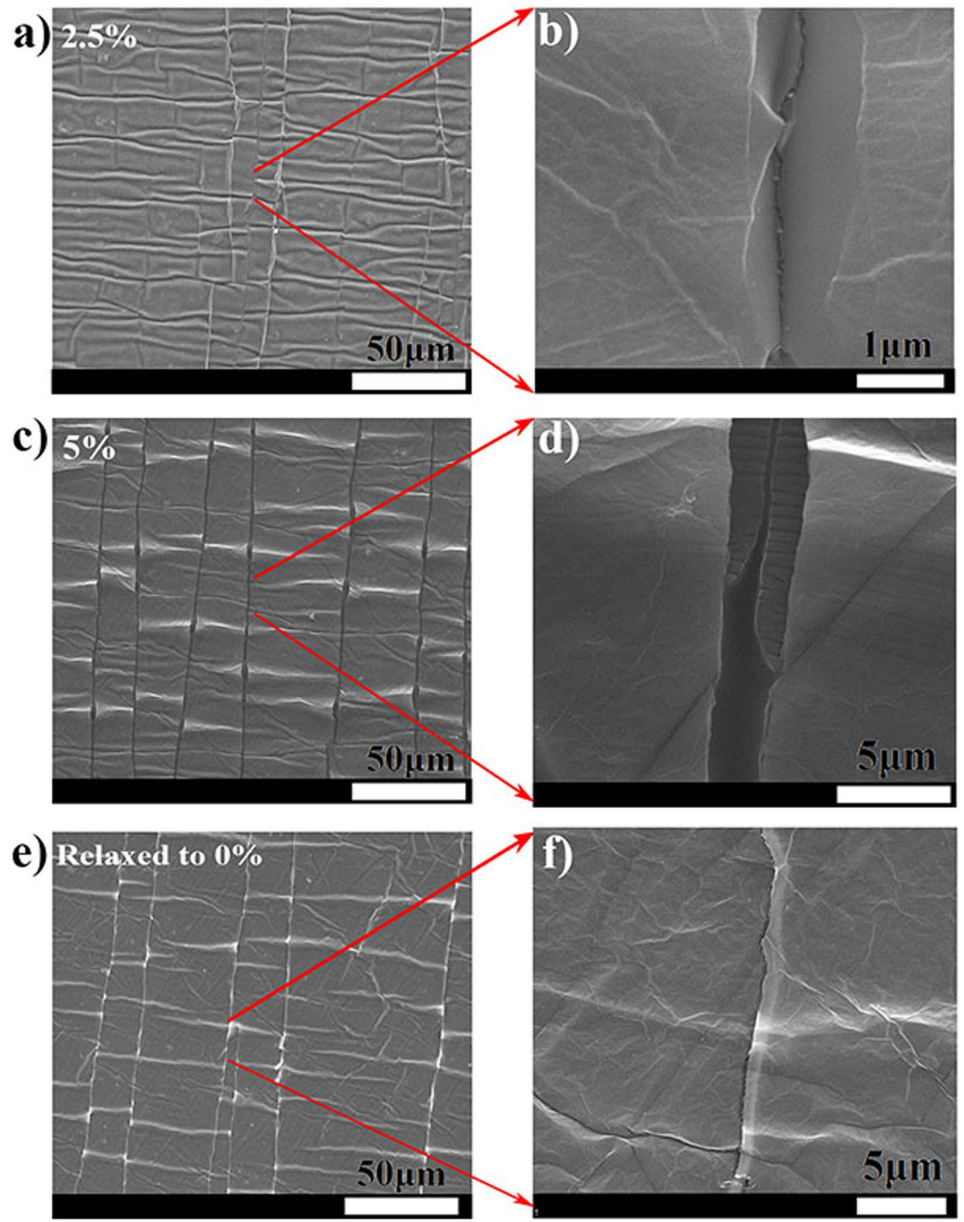
SEM images of 3-coat rGO-PDMS films under (**a**) 2.5% (**c**) 5% (**e**) Relaxed from 5% to 0%. (**b**), (**d**) an (**f**) are the zoomed in versions respectively.

Figure 2a and b shows the SEM image of rGO film at 2.5% applied strain illustrating that cracks are formed, but they do not cover the entire width of the sample. However, when a 5% strain is applied, quasi-periodic cracks are formed as shown in Fig. 2c, and the cracks are found to be propagating across the entire width of the sample. These quasi-periodic cracks in case of 5% strain have an average width of 3 μm (Fig. 2d). Figure 2e shows the SEM image of rGO films relaxed from 5% to 0%. After relaxation, due to its layered morphology, rGO sheets drape the crack partially in some areas and completely in other (Fig. 2f).

**Figure 3:**
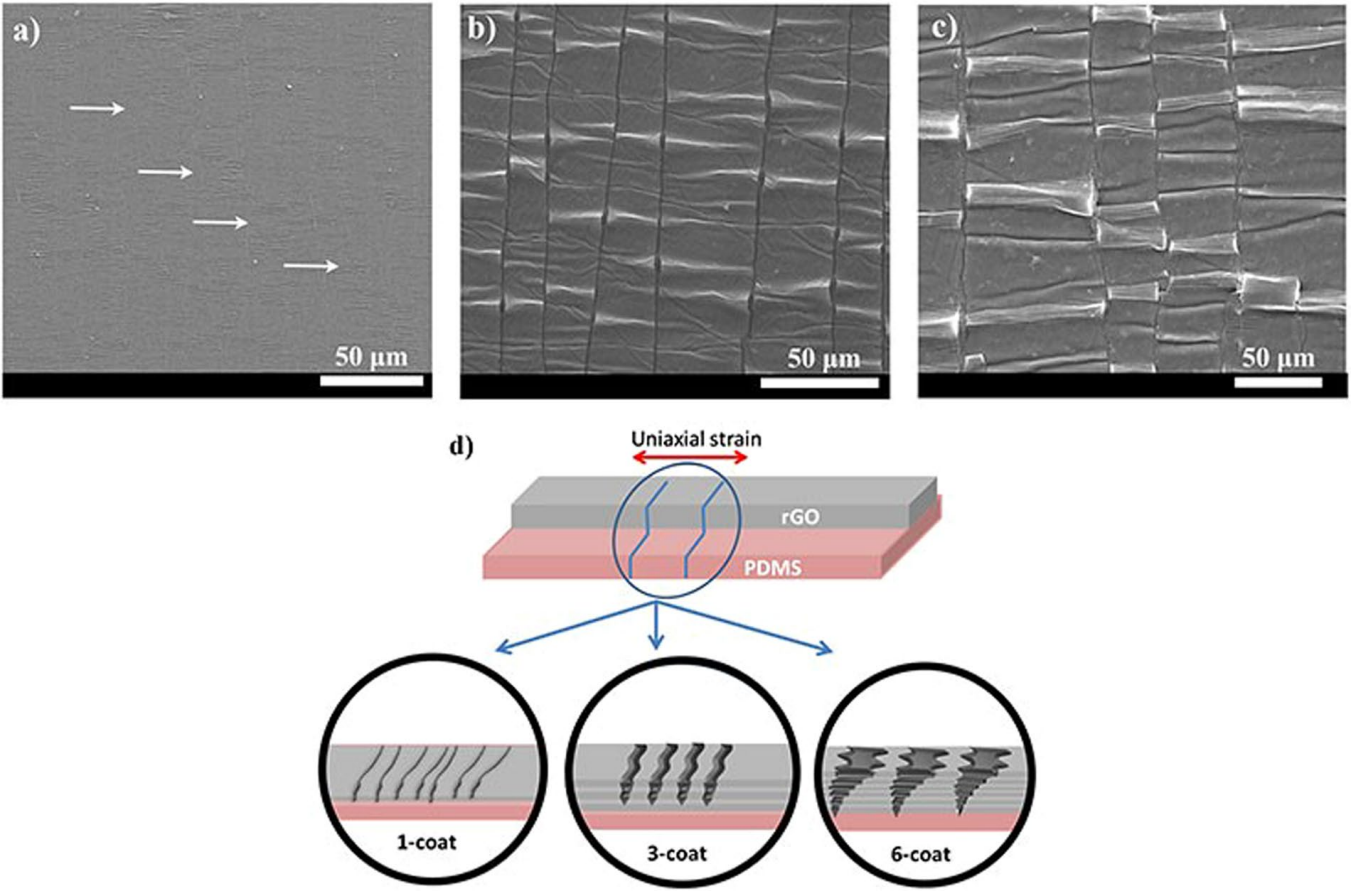
SEM images for (**a**) 1-coat (arrows indicate the position of cracks) (**b**) 3-coat (**c**) 6-coat rGO films on PDMS substrate at 5% applied strain. (**d**) Schematic diagram to illustrate the thickness dependent crack formation in rGO films under uniaxial strain.

We next consider the formation of cracks in uniaxially strained rGO films as a function of thickness. Film thickness plays a major role in defining the cracking process for thin films deposited on flexible substrates as suggested by various reports on different materials^34,35^. Figure 3 shows the SEM images of 1-coat, 3-coat, and 6-coat rGO-PDMS films respectively under 5% strain. The approximate thickness values for these films are mentioned in Table 1. It is observed in Fig. 3a to c that as the number of coats increases, the crack width increases from 280 nm to 3.5 µm. On the other hand, the crack density follows an opposite trend, with corresponding decrease of 4–5 times. This trend is schematically represented in Fig. 3d and quantified in Table (1).The crack density follows the same tendency irrespective of the straining rate. An important observation to be made here is that the number of cracks also depends on the strain rate. Larger strain rates generate more cracks (Table (1)). The strain rate dependence is commonly observed in thin films^36^ and also in polymer composite of which rGO is a component^37^. In a recent study on GO^38^, it has been demonstrated experimentally and theoretically that as the strain rate is lowered, interlayer interaction leads to stick-slip movement in GO layers. This stick-slip movement gives rise to strain controlled plastic behavior. These studies overall indicate that as the strain rate is lowered, the hardness of the film reduces^37^ and therefore due to an increase in ductile mechanism of strain energy release, material failure in the form of cracks is less prominent. This is reflected in our results indicating low density of cracks as the strain rate is reduced. However, importantly, a consistent thickness-dependence is observed independent of strain rate in our case. For example, the number of cracks per unit length for 1-coat is typically 4–5 times that for 6-coat, both at fast and slow rates of strain.

**Table 1:** Comparison of crack density and average crack width for 1, 3 and 6-coat rGO-PDMS films upon 5% of applied strain.

Number of Coats	Thickness (nm)	Crack density (per unit mm) with ramp rate of 0.0067% sec^−1^	Crack Density (per unit mm) with ramp rate of 0.5% sec^−1^	Average Crack Width (μm)
1	135	22	85	0.28 ± 0.02
3	175	14	42	1.94 ± 0.48
6	300	4	17	3.47 ± 0.52

To understand the cracking process in detail, we studied the progressive formation of cracks as a function of applied strain for different thickness. Figure S3 show the optical images of a representative 6-coat sample under the application of strain from 0 to 5%. The first notable feature about cracking is that there is a critical strain value (~3.6%), after which the cracks begin to appear. Two modes of cracking have been described in the literature: (i) sequential cracking and (ii) simultaneous cracking^39^. The former is observed in our samples, since the crack density monotonically increasing with strain beyond the critical strain value. The optical images also reveal new cracks forming between existing cracks, confirming the sequential cracking.

The thickness dependence of crack density can be understood in terms of the differences in the elastic moduli of the films and the substrate. PDMS has a Young’s modulus of *E*_*s*_ = 3 MPa and Poisson ratio of *v*_*s*_ = 0.45–0.5^40^, whereas, rGO is much stiffer with *E*_*f*_ = 185 GPa and *ν*_*f*_ = 0.149^41^. The large mismatch and further *E*_*f*_ ≫ *E*_*s*_ results in the Dundur parameter^39^

1 $${\rm{\alpha }}=\frac{{\bar{E}}_{f}-{\bar{E}}_{s}}{{\bar{E}}_{f}+{\bar{E}}_{s}}\sim 1$$

where $\bar{E}={\rm{E}}/(1-{\nu }^{2})$. Therefore the reference length ‘l’ which is related to Dundur parameters α and β, as

2 $$l=\sqrt{\frac{{\bar{E}}_{f}\times h}{k}}=\frac{\pi \,}{2}g(\alpha ,\beta )h$$

This equation (2) gives the thickness dependence *l*(*h*), while the material parameters are contained in the function, *g*(*α*, *β*). Sequential cracking can be described in terms of energy release rate ‘*G*’, at each advancing crack tip. The criteria *G* = *Γ*_*c*_, sets the condition for propagation of crack across the brittle film, where *Γ*_*c*_ is the fracture toughness of the rGO film. The solution for this problem has been obtained numerically in terms of film thickness ‘*h*’, elastic mismatch between the film and the substrate (contained in *l*), and crack spacing *H*. A detailed discussion in this regard is presented in S4, but it would suffice to note that the energy release rate in this problem, *G*, rapidly decreases as a function of *g*(*α*, *β*)*h* ^2^/*H*. So, thicker films with larger value of ‘*h*’ are associated with larger crack spacing ‘*H*’ to ensure that crack propagation can advance. In our samples, the term *h* ^2^ increases by a factor ~4–5 (from 1-coat to 6-coat). At the same time, crack spacing *H* also increases by a factor of ~4–5, which provides for the quantitative explanation. This factor is consistently observed for both slow and rapid ramp rates (see Table 1).

**Figure 4:**
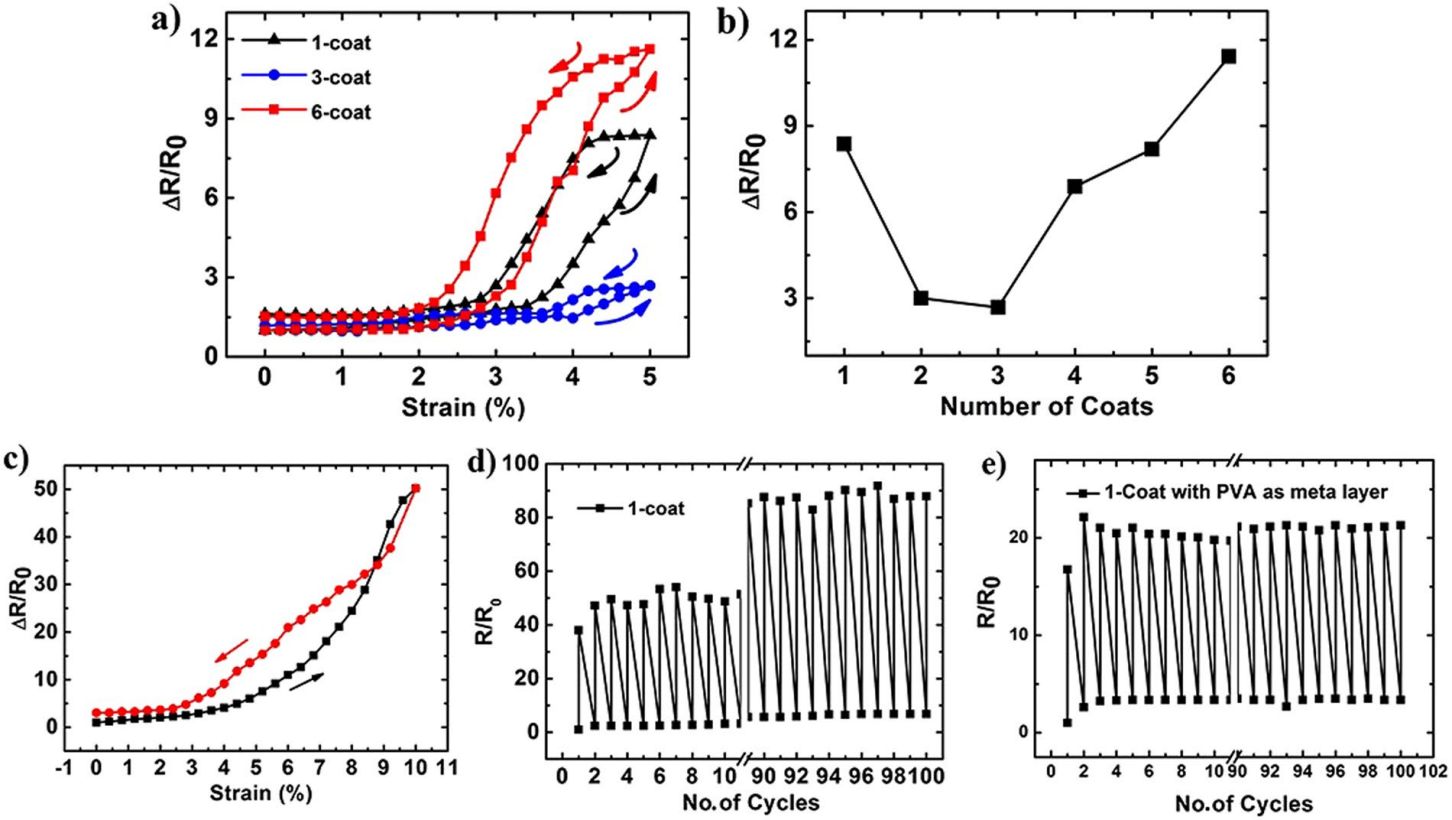
(**a**) Fractional change in resistance vs. strain for 1-coat, 3-coat and 6-coat films. (**b**) Resistance change of rGO films at 5% strain normalized to the resistance at 0% strain value vs. number of coats. (**c**) Strain response of 1-coat sample for up to 10% strain. Resistance vs. strain behavior of 1-coat sample up to 10% strain under cyclic loading test (100 cycles): (**d**) without meta-layer; (**e**) with PVA meta-layer.

So far we have discussed the morphology of crack formation at different values of uniaxial strain, and also the dependence of this phenomenon on film thickness. These morphological changes can also be expected to strongly influence the electrical response under strain. With this motivation, we prepared six kinds of samples ranging from 1-coat to 6-coat to evaluate the strain response as a function of thickness of rGO on PDMS substrate. The uniaxial strain was applied using a linear micro-manipulator stage. Figure 4a shows the variation of normalized resistance for 1-coat, 3-coat and 6-coat samples as a function of strain. For 1-coat sample, the resistance takes-off at an applied strain value of 3.5% and shows an eight fold increase in resistance for maximum strain value of 5%.

The reason for such an abrupt increase in resistance of these samples after particular (or critical) strain value can be attributed to the loss of inter-flake connectivity of rGO. This effect is evident from the 1-coat sample when released back to 0% strain, ΔR/R_0_ does not come back to its initial resistance, and it shows hysteresis behaviour. This also indicates that for a 1-coat sample, there is a permanent change in the structure of the film upon cycling. The strain response for 3-coat sample is characterised by low hysteresis nature and regains its initial resistance value when relaxed. For 3-coat sample, it takes ~4.2% of strain to show an abrupt change, which is ~2.5 times the initial value of 2.3 kΩ. It is evident that the 3-coat samples not only have the smallest size of the hysteresis loop, but also the smallest fractional change in resistance and highest critical value of strain for the onset of change in resistance. All these point toward the crack resistant nature of 3-coat films when compared to thicker or thinner films. This observation gains significance when considered with the fact that flexible transistors with conventional electrodes typically experience a strain with a maximum limit of 1.5 to 2%^11^. This suggests the scope of using 3-coat rGO films as electrodes for flexible devices with high scalability and ease of fabrication.

In order to explore if the increase in number of coats further enhances the stability towards applied strain, 6-coat sample was put under a similar straining/relaxation cycle as shown in Fig. 4a. It can be seen that resistance does not change beyond 1.5 times of initial resistance for applied strain values up to 2.6%. However, as the sample is strained beyond this value, a dramatic rise in the resistance is observed and it rapidly reaches to ~12 times the initial resistance value. As the thickness of the sample increases, strain energy required for crack formation and propagation is considerably reduced. The progressively larger change in fractional resistance of samples thicker than 3-coat, can be attributed to the progressive increase in crack width (Table 1). While both 1-coat and 6-coat samples show large changes in fractional resistance, release of strain in the latter restores the resistance to pre-strain values. This process is consistent with the observation of relaxation of cracks that happens upon release of applied strain, as discussed in the context of SEM images of strained and relaxed GO. Figure 4b shows the variation in resistance for rGO films with different number of coats at an applied strain value of 5%. As can be seen from the Fig. 4b, the value of ΔR/R_0_ decreases and reaches a minimum value for 3-coat. Further, increase in the number of coats leads to an increase in the value of ΔR/R_0_. For 3-coat sample there is an optimum balance between the crack density and crack width, neither of which are too high (Table 1) and in our transport experiments 3-coat films show the least change in the fractional resistance upon straining. To determine the strain response of rGO at strains >5%, 1-coat sample was put under strain in steps of 0.4% and the response is recorded as shown in Fig. 4c. It is observed that there is a steep rise in resistance after 5% strain and at 10% strain, resistance increased to ~50 times of its original value. The gauge factor thus calculated for 1-coat at 10% strain is found as 466. The gauge factor is limited by the layer-by-layer structure of rGO film, such that even if a single layer has lost its inter-flake connectivity, there is a relatively higher probability of existence of an alternate percolation path for conduction via interlayer transport and this helps in retaining its conductance even after the application of 5% strain. The flake size of rGO is 2 ± 0.5 μm. Upon sonication for 3 h, the size got reduced to an average value of ~250 nm (Figure S5). When the 1-coat sample is prepared with GO having smaller flake sizes, a gauge factor of ~1500 is realized, albeit with poor resistance recovery (Figure S6). This suggests that when the flake size becomes smaller, the inter-flake connectivity loss under strain results in larger change in resistance. Thus, a significantly large change in resistance observed for 1-coat and higher number of coats (n > 3) indicates their potential for strain sensing applications. In order to test the stability of the strain sensor, cyclic loading test for 1-coat sample is conducted. Figure 4d shows the resistance change at 10% strain for 1–10 and 90–100 cycles. The gauge factor obtained here is comparable with the recent report on a similar layered system^42^. A variation in fractional resistance change is also observed during the 100-cyclic loading test. This suggests that some part of the cracking and deformation of rGO is not reversible when the film is put through extended cycles of strain. In our work, we considered introducing polyvinyl alcohol (PVA) as a meta-layer for the rGO strain sensor. A plot of the cyclic loading test for 1-coat rGO on PDMS substrate with an intervening layer of PVA meta-layer is shown in Fig. 4e. The introduction of the meta-layer prevents the permanent deformation of rGO layers by stress-redistribution mechanism^26,35^ between the meta-layer and rGO. This makes the strain sensor more robust when the substrate is strained to high value of 10% over 100 cycles. With significantly diminished drift in the gauge factor across the 100 strain cycles, a gauge factor of ~270 is realized for the system when the PVA meta-layer is incorporated. When compared to the reports on stretchable composites^5,6^ we obtain a higher gauge factor. Therefore, we find that our work presents a strategy that can help to resolve the limitations of (i) low stretchability of layered films^42,43^ which have material failure at low strain values, and (ii) low gauge factor offered by several composite architectures^5,6^; which are the two commonly adopted strategies for flexible strain sensor.

**Figure 5:**
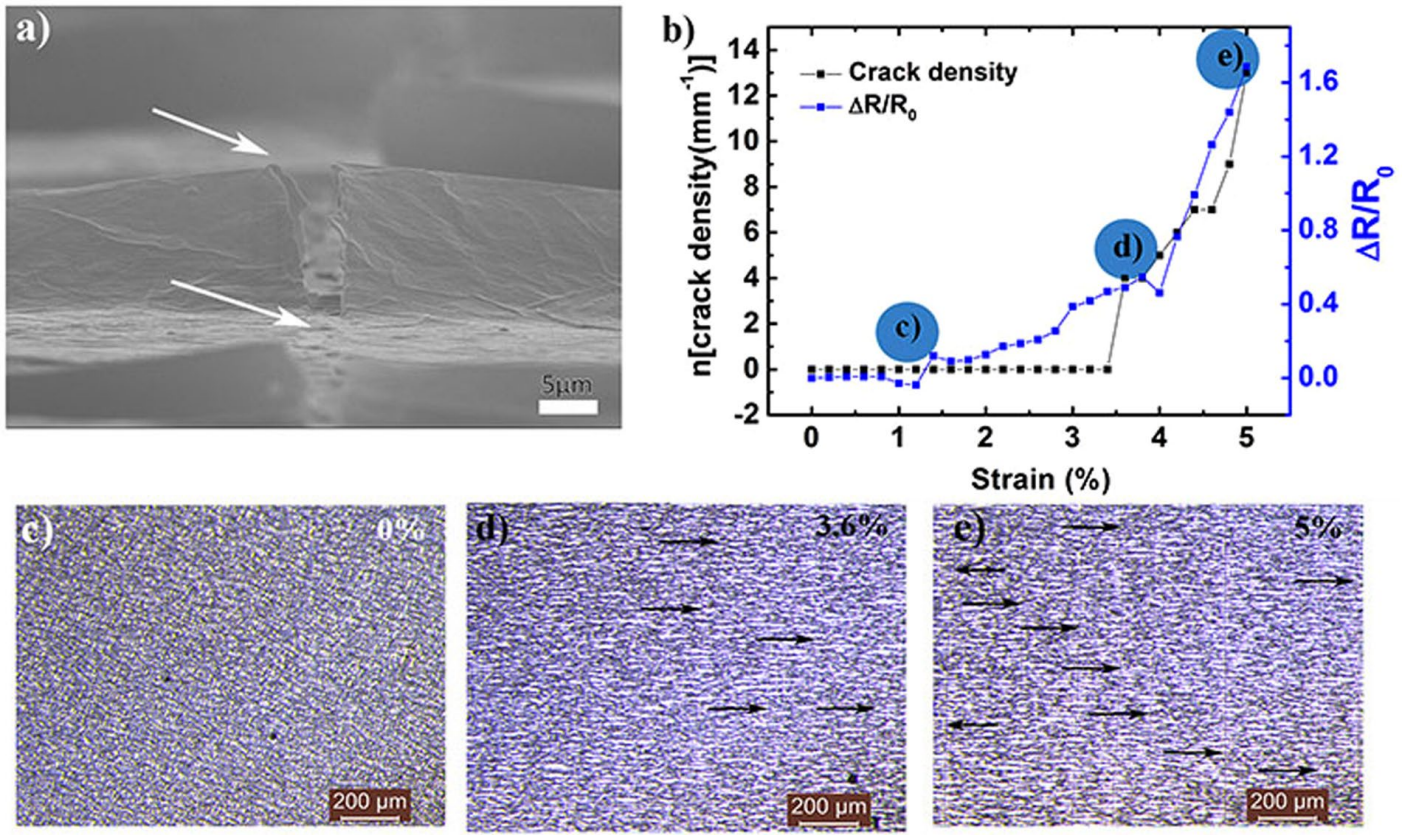
(**a**) Cross-section SEM image of 3-coat sample under 5% strain. The image was obtained after resolving the crack at extended depth of focus. The two arrows indicate the crack formation across the buckle and in the planar region of the sample. (**b**) Variation in crack density (n) and fractional resistance as a function of strain. (**c**,**d** and **e**) Optical images of 3-coat sample at 0, 3.6 and 5% applied strain, respectively. Blue labeled circles in Fig. 5(b) correspond to the optical images shown in (**c**–**e**).

To get more insight into this change in resistance upon straining, a detailed study was conducted which is discussed as follows. Figure 5a shows the cross-section SEM of 3-coat sample under 5% strain. Arrows point towards the crack formation in both buckled and planar region of the sample. To further investigate the effect of cracks on fractional resistance change as the strain is varied for 3-coat sample, variation in crack density (n) was studied as a function of applied strain shown in Fig. 5b. Both the fractional resistance and crack density vary exponentially after a critical strain value (~3.6%) is breached. The similar response of both parameters arises since crack density determines the fractional resistance change. The variation in crack density as a function of strain is shown in the representative optical images in Fig. 5c,d and e at 0, 3.6 and 5% strain, respectively.

**Figure 6:**
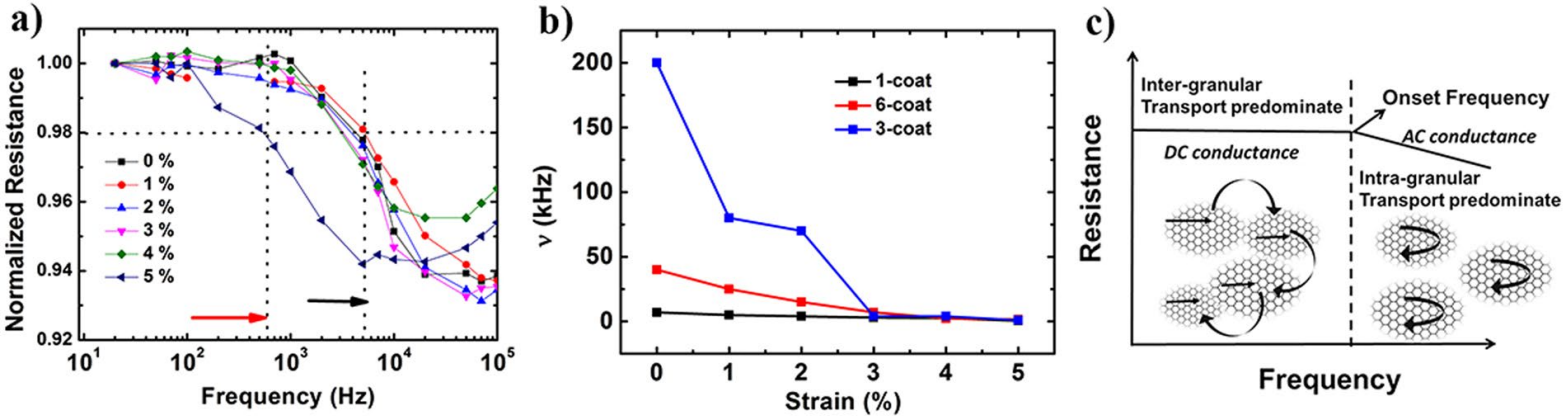
(**a**) Frequency response of 1-coat rGO-PDMS film under different strain values (red arrow marks the onset frequency for 5% of applied strain and black arrow marks the onset frequency for all other lower strain values). (**b**) Variation of onset frequency as a function of applied strain for rGO films with different number of coats. (**c**) Schematic to illustrate the dominant mode of frequency-dependent transport, before and after the onset frequency.

We next discuss the frequency-dependent ac conduction of these films as a function of strain. Figure 6a illustrates the Bode plot of resistance change in a representative 1-coat rGO-PDMS sample as a function of frequency (plotted on a logarithmic scale), for different values of applied strain. Consistent with the dc transport behaviour, it is observed that the ac resistance increases with applied strain. Further, after a particular frequency value the resistance starts decreasing from its dc value, which defines the onset frequency of ac conduction. This onset frequency is found to vary as a function of strain. Similar experiments to study frequency response as a function of thickness are conducted for 3-coat and 6-coat; from these plots, the values of the onset frequency are deduced.

Comparison of the variation in onset frequency for 1, 3 and 6 coat is shown in Fig. 6b. For 3-coat sample, ac conduction marked by onset frequency starts at 200 kHz at 0% strain and it is found to be 40 kHz and 7 kHz for 6-coat and 1-coat respectively. It can be seen in general for the three samples that onset frequency shifts to lower values at a critical strain value of 4–5%, which coincides with the emergence of quasi-periodic cracks in the film. The onset frequency for ac conduction is an important figure of merit for disordered conducting films^44^. Figure 6c shows the schematic representation of the variation in the dominant mode of transport before and after the onset frequency. In a granular conducting media, ac conduction is contributed by both inter-grain and intra-grain transport and varies as a function of frequency^44^. Before the onset frequency, the conduction is dominated by inter-grain transport, while after the onset frequency intra-grain is the major contributing process which leads to a decrease in resistance. The onset frequency in ac transport is related to the size of conducting islands in the granular medium^33^. Consistent with the DC measurements shown Fig. 4, there is a major increase in resistance value for an applied strain value >4% strain. This rapid increase in resistance is also reflected in the onset frequency variation with applied strain which suggests that after 4% strain there is a high disruption in the percolation paths between the conducting portions of different rGO grains. The value of critical strain for 3-coat sample as per the linear elastic fracture mechanics model^45^ is found to be ~4.7% which is very close to the experimental data observed.

So far we have demonstrated that very thin (1-coat) or thick (6-coat) films can be adapted for strain sensing. The intermediate thickness (3-coat) shows the least response to applied strain and is therefore suited in applications like flexible optoelectronics, where strain-related effects are not a desirable feature. To discuss the role of thickness-dependent crack propagation in a strained optoelectronic device, we prepared a hybrid of a standard UV active material - TiO_2_ nanoparticles with rGO on flexible PDMS substrate.

**Figure 7:**
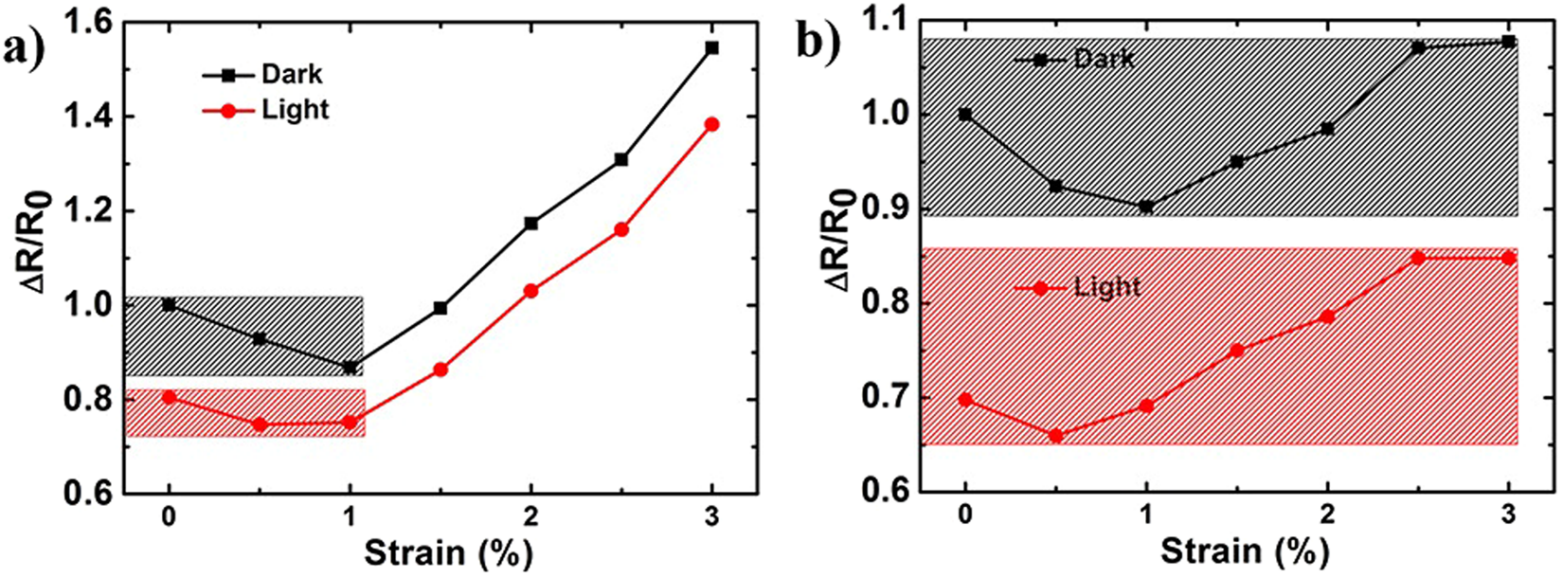
Optoelectronic response under strain for rGO-TiO_2_ hybrid system for (**a**) 1-coat and (**b**) 3-coat films. The shaded regions indicate device operation windows where photodetection is possible even in the presence of strain, without incorporating any strain-dependent corrections.

Figure 7a illustrates the UV response of TiO_2_-rGO system with 1-coat rGO as electrode. As can be seen from figure, the resistance under dark and light conditions continues to increases as a function of applied strain and does not show a stable response. For example, the strain dependence of dark current implies that the change in resistance of a flexible photodetector can be attributed either to the strain and its associated crack propagation or to the generation of photocarriers. The device, when used in the flexible configuration, is not capable of distinguishing these two factors since both influence the resistance. Figure 7b shows the response of TiO_2_-rGO system with 3-coat rGO as electrode. Both the dark resistance and resistance under illumination have a much weaker dependence on strain. For this case, a change of resistance of the device can directly be attributed to photodetection. Thus optimization of rGO thickness is crucial for applications which require resistance to strain.

## Conclusions

In summary, we present a systematic study on the strong thickness-dependence of crack propagation in sub-micron thick rGO films under uniaxial strain. Morphological investigations reveal the formation of quasi-periodic cracks with crack density decreasing with thickness, while the crack width shows an increase with thickness. Varying the thickness of the films, therefore, provides a knob to tune the strain response of the films, which is important for tailor-made strain-sensing applications. The same material can be made strain-resistant by optimizing the thickness, and the effects of this are also seen in strain-dependent electrical conductivity studies and further in rGO-TiO_2_ hybrid optoelectronic devices strained on flexible substrates. The concepts explored here can be generalized to a wide variety of layered 2D systems, their derivatives and hybrids towards the design of a variety of applications ranging from strain sensors to flexible optoelectronic devices.

## Materials and Methods

Graphene Oxide (GO, 6 mg/ml aqueous dispersion from Graphene Laboratories) was further diluted by adding equal amounts (vol/vol %) of ethanol to obtain 3 mg/ml GO dispersions in ethanol-water mixture. GO on flexible substrate was prepared by spin coating GO dispersions in ethanol-water mixture on PDMS (Polydimethylsiloxane) thin films. PDMS film (~0.5 mm) was moulded on a glass substrate by spin coating and then baked at 80 °C for 1 h. PDMS surface was made hydrophilic by oxygen plasma treatment. Solution of GO (3 mg/ml) in ethanol-water mixture, was then spin coated using a three-step process at varying speeds: 500 rpm −30 s; 800 rpm −60 s and 1500 rpm −60 s. GO films were annealed at 180 °C for 30 minutes in Ar atmosphere for reduction. rGO-PDMS films were peeled off from the substrate after cool-down to get flexible free standing films. rGO-PDMS films with different thickness were prepared by successive spin coating of GO dispersion and further annealing. The samples were named upon the number of times spin coating is performed over them, for example 1-coat samples implies spin coating was performed only once. To prepare PVA meta-layer based rGO-PDMS films, 1% PVA aqueous solution was spin coated onto O_2_ plasma treated PDMS films. Thereafter, GO solution was spin coated, followed by annealing procedure as discussed earlier. Structural characterization of GO and rGO was done using Raman spectroscopy, XRD and XPS. Optical Characterization was performed using Leica Stereozoom microscope (M205 C). The thickness measured using Optical Surface profilometry (Bruker, Non-contact) was found to be ~135, 150, 175, 200, 300 and 300 nm for 1-, 2-, 3-, 4-, 5- and 6-coat samples, respectively. Addition of an extra coat beyond 5-coats using successive spin-coating technique did not increase the thickness, and hence experiments with additional coats of GO beyond 6-coat films were not considered. Morphology of rGO film is characterized by TEM (Joel 2100) and SEM (FEI Quanta FEG 200). Flake size of GO is determined by spin coating 0.1 mg/ml on SiO_2_/Si substrate and dried sample is imaged by Raith 150^TWO^ system electron beam lithography. For electrical measurements, 4-terminal in-line contacts were used. Signal generation and voltage sensing was done with SR830 DSP lock-in amplifier at low frequency (13.5 Hz). For frequency-dependent ac conduction, two-terminal samples were used. Frequency-dependent electrical measurements were performed using an Agilent E4980A LCR meter. Standard compensations for open and short circuit corrections were included, while the geometry of contacts was two probes. Strain measurements were performed by mounting the as-prepared rGO-PDMS sample on a linear manipulator stage and uniaxial tensile strain in steps of 0.2% was applied. For optoelectronic studies under strain, a hybrid of TiO_2_-rGO on PDMS was prepared, as described below. TiO_2_ nanoparticles (NPs) were dispersed in rGO (3 mg/ml) and then the solution was sonicated for 1 h. The resulting solution of rGO-TiO_2_ NPs was then spin coated on the oxygen plasma treated PDMS films once to make 1-coat sample and thrice to make 3-coat sample. The samples were then exposed to UV lamp (365 nm) of 5 mW power and strain was applied in steps of 0.5% using the micromanipulator. Response was recorded for each strain value under both dark and light conditions.

## Electronic supplementary material

Supporting Information
