# Erratum: Thickness-dependent Crack Propagation in Uniaxially Strained Conducting Graphene Oxide Films on Flexible Substrates

**DOI:** 10.1038/s41598-017-09614-2

**Published:** 2017-11-01

**Authors:** Tushar Sakorikar, Maheswari Kavirajan Kavitha, Pramitha Vayalamkuzhi, Manu Jaiswal

**Affiliations:** 1 Department of Physics, Indian Institute of Technology Madras, Chennai, 600036 India; 2 Department of Electrical Engineering, Indian Institute of Technology Madras, Chennai, 600036 India

*Scientific Reports* **7**:2598; doi:10.1038/ s41598-017-02703-2; Article published online 01 June 2017

In the PDF version of the original Article the text that appeared on page 4 should have appeared on page 5 and vice versa due to a typesetting error. This has now been corrected in the PDF version of the Article; the HTML version was correct at the time of publication.

In addition, the previous version of this Article contained an error in the ‘Results and Discussions’ section.

“To understand the cracking process in detail, we studied the progressive formation of cracks as a function of applied strain for different thickness. Figure S3 show the optical images of a representative 6-coat sample under the application of strain from 0 to 5%. The first notable feature about cracking is that there is a critical strain value (~3.6%), after which the cracks begin to appear. Two modes of cracking have been described in the literature: (i) sequential cracking and (ii) simultaneous cracking^39^. The latter is observed in our samples, since the crack density monotonically increasing with strain beyond the critical strain value. The optical images also reveal new cracks forming between existing cracks, confirming the sequential cracking.”

now reads:

“To understand the cracking process in detail, we studied the progressive formation of cracks as a function of applied strain for different thickness. Figure S3 show the optical images of a representative 6-coat sample under the application of strain from 0 to 5%. The first notable feature about cracking is that there is a critical strain value (~3.6%), after which the cracks begin to appear. Two modes of cracking have been described in the literature: (i) sequential cracking and (ii) simultaneous cracking^39^. The former is observed in our samples, since the crack density monotonically increasing with strain beyond the critical strain value. The optical images also reveal new cracks forming between existing cracks, confirming the sequential cracking.”

This error has now been corrected in the PDF and HTML versions of the Article.

