# Supplementary material for: Thickness-dependent Crack Propagation in Uniaxially Strained Conducting Graphene Oxide Films on Flexible Substrates
Source: Sci Rep. 2017 Jun 1;7:2598. doi: 10.1038/s41598-017-02703-2 (PMC5453986; doi:10.1038/s41598-017-02703-2)
Supplement: Supplementary file 1 — Supporting Information [file 41598_2017_2703_MOESM1_ESM.pdf]

Supporting information

Thickness-dependent Crack Propagation in Uniaxially Strained Conducting Graphene Oxide Films on Flexible Substrates

Tushar Sakorikar[1,2‡], MaheswariKavirajan Kavitha[1‡], Pramitha Vayalamkuzhi[2,*], and Manu Jaiswal[1,*]

[1]Department of Physics,Indian Institute of Technology Madras,Chennai – 600036, India

[2]Department of Electrical Engineering,Indian Institute of Technology Madras,Chennai – 600036, India

[‡]MKK and TS contributed equally to this work

[*]Email: manu.jaiswal@iitm.ac.in, pv@iitm.ac.in

S1. Formation of wrinkle patterns in rGO films on PDMS substrates

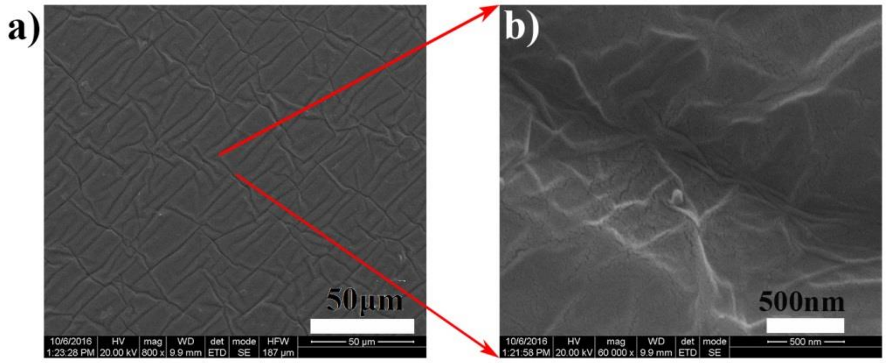

Figure S1.(a) SEM images of 3-coat rGO sample 0 % strain. **(b)** SEM image taken in magnified scale to show the sub-micron wrinkles in unstrained rGO.

The wrinkling wavelength for a 2-layer system, uses the following equation:[1]

$$\lambda = 2\pi h \left(\frac{(1-\nu_s{}^2)E_f}{(1-\nu_f{}^2)E_s}\right)^{\frac{1}{3}}$$

Where E is Young's Modulus and ν is Poisson's Ratio (The subscripts f and s denote the film and substrate, respectively)

The system studied in our work actually involves a 3-layer geometry. The top layer is rGO, the interfacial layer is a thin oxide produced during the oxygen plasma treatment of PDMS, while the substrate is bulk PDMS. Despite the complexity of having a 3-layer system, rather than a 2-layer system, we can consider the wrinkle formation due to mismatch in TEC for the interface formed by the rGO: oxidized layer as well as the interface formed by the oxide layer: bulk PDMS. For the rGO: oxidized layer interface, plugging the values of E_s= 790 MPa, ν_s= 0.45 – 0.5 and E_f = 185 GPa, ν_f = 0.149 and h = 175 nm, we obtain the value of λ ~ 4.5 μm for the rGO wrinkles. Note that the oxidized layer has much larger Young's Modulus than bulk PDMS and we have used the values based on the work of Bowden et al.[1] Similarly, for the oxide layer: PDMS interface, we use the elastic parameters of bulk PDMS as the substrate, the wavelength of wrinkles in the oxide layer will be λ ~ 7.5 μm.[1] Thus the wavelengths of wrinkles could be expected in the range of 4.5-7.5 μm. The experimentally observed λ obtained from the SEM images (Figure S1 a) ~ 3-12 μm, which is quite comparable to the numerical estimates.

S2. Wrinkle relaxation under strain

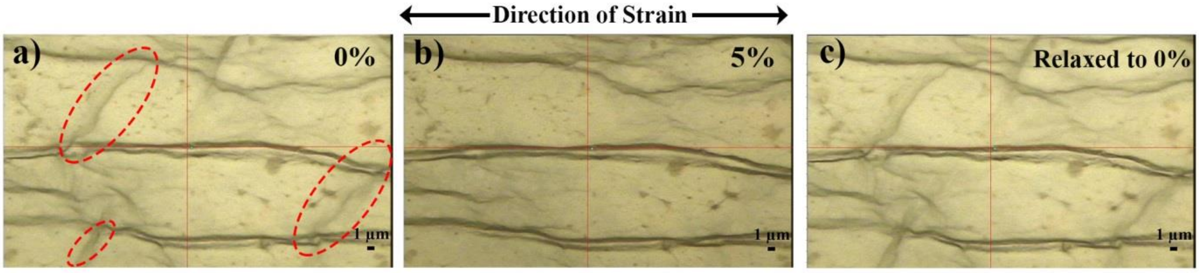

Figure S2.(a), **(b)**, and **(c)** Optical images of rGO film on PDMS substrate for strain values of 0 %, 5 % and then released back to 0 % respectively.

When a uniaxial strain is applied on rGO films, these wrinkles play a critical role in resisting the deformations of films. Figures S2a to S2c illustrate the relaxation of wrinkles under strain and recovery of wrinkles when strain is completely removed. In Figure S2a, the encircled regions are the wrinkles in rGO films transverse to the direction of applied strain. As the strain is increased to 5%, these wrinkles flatten completely. When the sample is relaxed back to 0% strain, the wrinkles reappear demonstrating a reversible nature of the morphology of rGO films. Wrinkles that are parallelto the direction of applied strain cannot dissipate their stored energy and hence their profile remains unchanged. Wrinkles that are transverse alone are able to relax and go to the minimum energy state by flattening, as the strain is increased. Relaxation of wrinkles provides resistance up to a certain value of strain, after which the crack propagation gets locally initiated.

S3. Sequential Crack Propagation

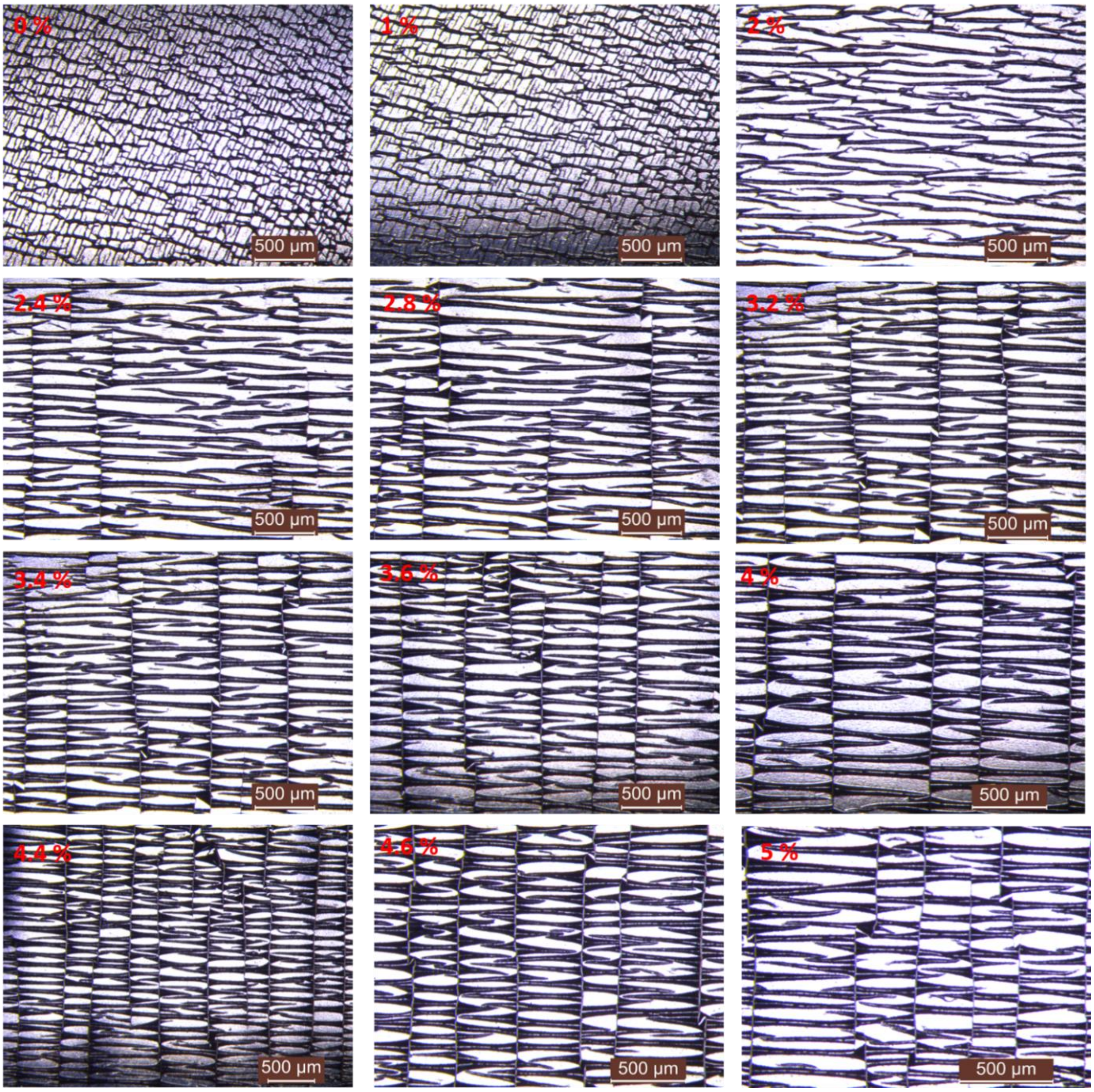

Figure S3. Optical image of 6-coat rGO film under the application of strain from 0 to 5 % showing crack formation

S4: Description of fracture mechanism

The energy release rate G at the crack tip for a semi-infinite isolated crack is given by[2]:

$$G = \frac{\pi}{2}\frac{(1-\nu^2)h\sigma^{0^2}}{E}g(\alpha,\beta) \qquad (1)$$

Where

$$\alpha = \frac{\bar{E}_f-\bar{E}_s}{\bar{E}_f+\bar{E}_s} \; and \; \beta = \frac{1}{2}\frac{\mu(1-2\nu_s)-\mu_s(1-2\nu_f)}{\mu(1-2\nu_s)+\mu_s(1-2\nu_f)}$$ are Dundur's parameters,

E is the Young's Modulus and ν is the Poisson's ratio (The subscripts f and s denote the film and substrate, respectively),

$\mu = E_f/(2(1+\nu_f))$and$\bar{E}_f = E_f/(1-\nu_f{}^2)$ denote the shear modulus and plane strain tensile modulus respectively, $\sigma^0$is the uniform prestress in the film acting normal to the crack line.

However, equation (1) is not sufficient to describe the formation of parallel semi-infinite cracks which can manifest in two modes:

(i) Sequential Cracking: Here a new crack is formed between two existing cracks, as the applied strain is increased.

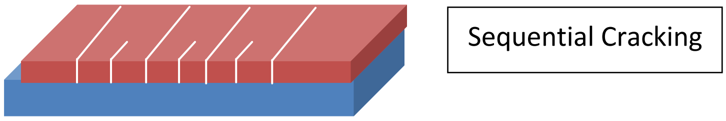

(ii) Simultaneous Cracking: Here the cracks form simultaneously upon the application of strain.

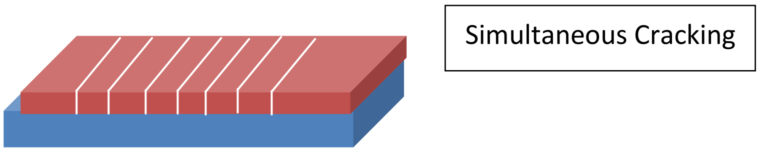

Figure S4. Schematics of Sequential versus Simultaneous mode of cracking.

In our case sequential cracking is observed (see main text) and therefore the equation governing the energy release rate is modified as[2]:

$$G = \frac{l\sigma^{0^2}}{\bar{E}_f}\left[2\tanh\left(\frac{H}{2l}\right) - \tanh\left(\frac{H}{l}\right)\right] \qquad (2)$$

Here G is the difference between the energy release rate at the crack tip in formation of first set of cracks and the crack tip for the next set of cracks that appear in between the existing cracks.

And $l = \sqrt{\frac{\bar{E}_f h}{k}} = \frac{\pi}{2}g(\alpha,\beta)h$, is defined as the reference length

Following is the schematic plot for two modes of cracking[2]

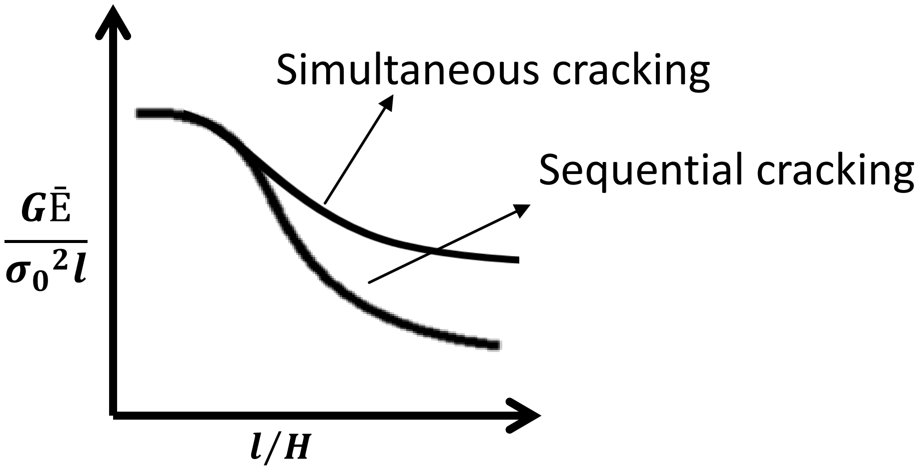

In our case since we observe sequential cracking, the following graph should govern the energy release rate as a function of thickness and crack spacing therein. Here, $\Gamma_c$is the mode-I fracture toughness

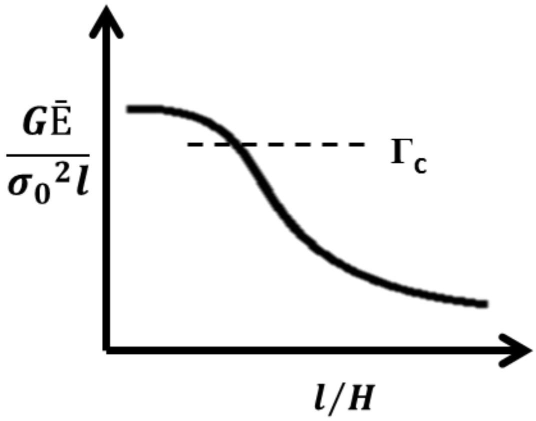

For a crack spacing $2H$, from equation (2), $G = \Gamma_c$ defines the condition for the channelling new set of cracks ideally halfway in between the existing cracks by the process of sequential cracking. In above graph, multiplying both X and Y-axes by 'l' (since multiplying both axes doesn't change the overall nature of graph) we get:

$$Gq \sim ph^2/H$$

Where $q = \frac{\bar{E}}{\sigma_0{}^2}$ and $p = g^2(\alpha, \beta)$

This square dependence of energy release rate with film thickness fits well in our case as the term 'h^2' increases by a factor ~ 4- 5 (from 1-coat to 6-coat). At the same time, crack spacing also increases by a factor of ~ 4- 5, which provides for the quantitative explanation. This factor is constantly observed for both slow and rapid ramp rates.

S5. Modification of rGO flake-size by sonication

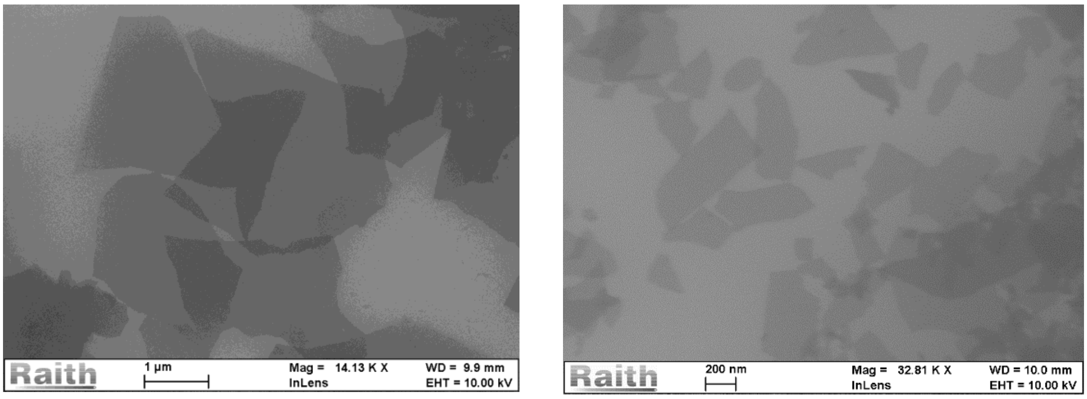

(a) **(b)**

Figure S5. SEM image of GO with (a) bigger flake size and (b) smaller flakes obtained after sonication for 3 h

S6. Response for smaller rGO flake size

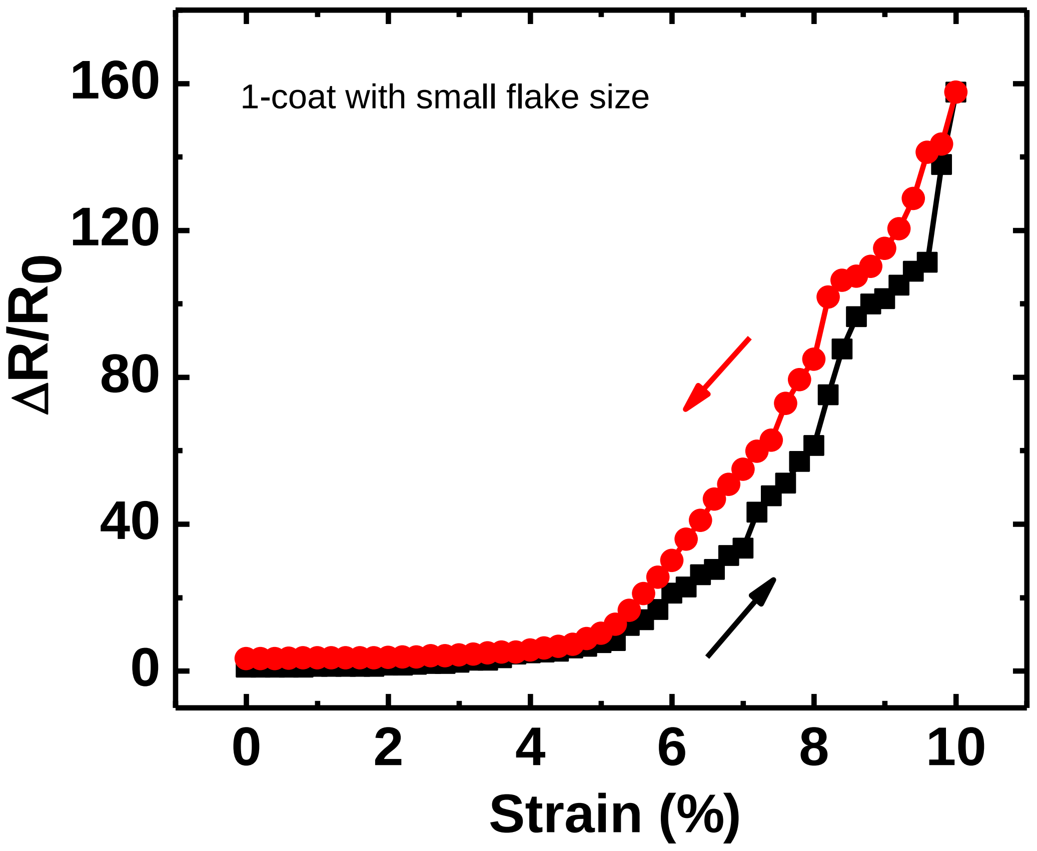

Figure S6. Fractional change in resistance vs. strain for 1-coat film prepared using smaller flake size GO

1 Bowden, N., Huck, W. T. S., Paul, K. E. & Whitesides, G. M. The controlled formation of ordered, sinusoidal structures by plasma oxidation of an elastomeric polymer. *App. Phys. Lett.* **75,** 2557 (1999).

2 Xia, Z. C. & Hutchinson, J. W. Crack patterns in thin films. *J Mech. Phys. Solids* **48,** 1107 (2000).
